# The effect of Non-Motor symptoms on Health-Related quality of life in patients with young onset Parkinson’s Disease: A single center Vietnamese Cross-Sectional study

**DOI:** 10.1016/j.prdoa.2021.100118

**Published:** 2021-11-24

**Authors:** Tai Ngoc Tran, Uyen Ngoc Le Ha, Tuan Manh Nguyen, Thuan Duc Nguyen, Khang Ngoc Chung Vo, Thuong Huyen Dang, Paula Mai Phuong Trinh, Daniel Truong

**Affiliations:** a Movement Disorder Unit, Neurology Department, University Medical Center, University of Medicine and Pharmacy, Ho Chi Minh City, Viet Nam; b Neurology Department, 103 Military Medical University, Hanoi, Viet Nam; c The Parkinson and Movement Disorder Institute, Fountain Valley, CA 92708, USA; d Department of Psychiatry and Neuroscience, University of California Riverside, Riverside, CA, USA

**Keywords:** Young onset Parkinson’s disease, Non-motor symptom, Quality of life

## Abstract

**Background:**

Young onset Parkinson’s disease (YOPD) is a distinct entity from typical late onset Parkinson’s disease (LOPD). The influene of non-motor features on the health - related quality of life (HRQoL) in LOPD has been previously reported, but little is known about the impact of non-motor features in YOPD.

**Objective:**

The aim of this study was to explore the relationship between non-motor burden and HRQoL in patients with YOPD.

**Methods:**

This was an observational, cross-sectional study in patients with a PD, whose age at disease onset ranged from 21 to 40 years (YOPD). Participants were assessed with the MDS Unified Parkinson’s Disease Rating Scale (MDS-UPDRS), Non-Motor Symptoms Scale (NMSS) and the 39-item Parkinson’s Disease Questionnaire (PDQ-39; range 0–100). Spearman’s rank test was used to identify correlations between NMSS domains and several dimension of HRQoL. Stepwise multiple linear regression analysis was performed to identify the independent predictors of HRQoL as measured by PDQ-39 summary index.

**Results:**

89 patients with YOPD mean (SD) age = 42.15 (5.84) participated. Patients reported 10.17 (4.74) non-motor symptoms, the most common (75%) and severe (median = 3) of which was was fatigue (IQR = 7). The most frequently reported and severely affected NMSS domain was sleep/fatigue (89.9%, median = 8; IQR = 13) followed by mood/cognition (83.1%, median = 6; IQR = 18) and attention/memory (82%, median = 5; IQR = 8). The mean (SD) summary index of PDQ-39 was 32.89 (16.8). The means (SD) of each PDQ-39 dimensions were: mobility 37.33 (21.96), ADL 42.93 (25.33), emotional well-being 39.77 (25.47), stigma 38.19 (28.44), social support 19.03 (22.89), cognition 29.59 (20.63), communication 26.96 (23.57), and bodily discomfort 29.96 (23.19). With the exception of gastrointestinal tract and sexual function, all other NMSS domain scores were correlated with the PDQ-39 summary index. The multivariate model revealed that three NMSS domains including sleep/fatigue, mood/cognition and attention/memory accompanied with UPDRS part III were independent predictors of HRQoL as measured by PDQ-39SI.

**Conclusions:**

Non-motor symptoms pertaining to sleep disturbances/fatigue, mood/cognition and attention/memory negatively impact HRQoL in patients with YOPD.

## Highlights

• Non-motor features may negatively impact those with young-onset Parkinson disease.
• Sleep/fatigue was the most severely affected, followed by mood/cognition.
• These domains independently predicted health-related quality of life (HRQoL).

## 1 Introduction

### 1.1 Background

Parkinson’s disease (PD) is a common progressive neurodegenerative disease with an average age of onset varying from 60 to 69 years [1]. In the literature, the term young onset Parkinson’s disease (YOPD) usually refers to PD with the early appearance of first motor symptoms occurring between 21 and 40 years of age [2], [3]. However, due to lack of a consensus, the upper limit of onset age for YOPD has been raised to 50 years in several studies [4], [5]. The prevalence of PD varies widely, ranging from 30 to 180 cases per 100,000 around the world [6]. The YOPD subtype is reported to account for about 5% of all PD cases in Western countries and up to 10% in Japan [7], [8].

YOPD has many similarities with more classical PD, including cardinal motor symptoms such as bradykinesia, rigidity and rest tremor [5], as well as non-motor symptoms including cognitive decline, mental problems, sleep disturbances and autonomic dysfunctions [9]. However, YOPD has several distinct characteristics as well. Compared to LOPD, YOPD is characterized by longer lifespan, slower disease progression, higher incidence of dystonia (both at the onset and throughout the disease course), less common cognitive impairment and greater tendency to develop levodopa induced complications, namely dyskinesia and motor and non-motor fluctuations [10], [11], [12], [13]. Therefore, the treatment approach for YOPD is quite different from that for LOPD [14].

PD is well known to lead to disability and diminished quality of life [15]. Reduction in health-related quality of life (HRQoL) has been reported in patients with both LOPD and YOPD [16]. Some studies have shown that the negative effects of PD on quality of life are more significant in patients with young-onset disease [17], [18].

Several studies in patients with typical-onset PD have reported that quality of life correlates with the severity and burden of both motor and non-motor symptoms [19], [20], [21], [22], [23]. However, little is known about the influence of non-motor symptoms on the quality of life in YOPD patients [24]. Therefore, this study aimed to determine the relationship between non-motor manifestations of PD and HRQoL in patients with young-onset disease.

## 2 Patients and methods

This was an observational, cross-sectional study.

### 2.1 Participants

Eighty-nine patients who were clinically diagnosed with idiopathic PD (based on the International Parkinson’s Disease and Movement Disorder Society 2015 Diagnostic Criteria [25]) with an age of onset between 21 and 40 years of age were successfully recruited from the Movement Disorder Clinic, University Medical Center Ho Chi Minh City, Vietnam. The recruitment period was from November 1, 2019 to July 30, 2020. Patients who were unable to read or understand the questionnaires or had comorbidities, sequelae, or any disorder that could interfere with the assessment were excluded from the study. The sample size was calculated based on the formula for correlation studies with an estimate of moderate correlation (R = 0.5) based on a previous study on the correlations between non-motor symptoms and quality of life in PD patients [26].

### 2.2 Assessments

All participants provided written informed consent. Information on socio-demographic and disease-related characteristics were collected. Participants were then assessed with the following instruments: Non-Motor Symptoms Scale (NMSS) [27], Movement Disorder Society-Unified Parkinson’s Disease Rating Scale (MDS-UPDRS) [28], and the Parkinson’s Disease Questionnaire-39 items (PDQ-39) [29]. The NMSS is comprised of 9 domains and 30 items: cardiovascular (two items), sleep/fatigue (four items), mood/cognition (six items), perceptual problems/hallucinations (three items), attention/memory (three items), gastrointestinal tract (three items), urinary (three items), sexual function (two items) and miscellaneous (four items), which were used to evaluate the severity of non-motor symptoms. Higher scores indicate greater difficulty with the associated item or domain, so that higher scores indicated lower HRQoL. The MDS-UPRDS is a validated PD-specific scale consisting of four parts: part I (non-motor experiences of daily living), part II (motor experiences of daily living), part III (motor examination) and part IV (motor complications). MDS-UPDRS part III was used to evaluate the severity of motor symptoms. The PDQ-39 is a self – report questionnaire assessing PD-specific HRQoL. It is composed of 8 dimensions and 39 items: mobility (10 items), activities of daily living (ADL; six items), emotional well-being (six items), stigma (four items), social support (three items), cognition (four items), communication (three items), and bodily discomfort (three items). The scales were assessed during the “ON” state of medication.

### 2.3 Statistical analysis

Data were analyzed with SPSS 20 for PC. For descriptive statistics, quantitative variables with normal distributions (based on Kolmogorov–Smirnov test) were reported by means and standard deviations (SD). Non-normally distributed variables were reported by medians and interquartile ranges (IQR). The frequency distributions of each NMSS item and domain were calculated as percents.

As for inferential statistics, the Pearson correlation coefficients, or the Spearman rank correlation coefficients in case of non-skewed data, were utilized to establish the individual correlations between HRQoL as measured by the calculated PDQ Summary Index (PDQ-39SI) and each NMSS domain. Potential correlations between HRQoL and several socio-demographic and disease related factors including age, age of onset, disease duration, L-dopa daily dose, H&Y stage and UPDRS were also explored. The significance across all analyses was assessed at alpha (α) = 0.05.

All statistically significant covariates and clinically relevant variables were entered into multivariate analysis to determine the probability of influence (if any). Using the PDQ-39SI as the dependent variable, a stepwise multiple linear regression was performed to identify the independent predictors of reduced HRQoL.

This study was reviewed and approved by the Ethics committee of the University of Medicine and Pharmacy, Ho Chi Minh City.

## 3 Results

### 3.1 Study population characteristics

A total of 89 patients including 47 males and 42 females enrolled in this study. Most of the patients (73%) were manual laborers and the rest (27%) were white collar workers. The majority (41.6%) had completed secondary school education; 29.1% had completed college or beyond. The mean age and the mean age of disease onset were 42.15 ± 5.84 years and 35.46 ± 3.96 years, respectively. The mean disease duration was 6.68 ± 4.48 years. Of the participants, 19.1% had at least one close relative with PD. Socio-demographic and disease-related characteristics are shown in Table 1.

**Table 1:** Demographic and disease - related characteristics of the study YOPD sample.

Characteristics	Overall (n = 89)
Sex	
Males Females	47 (58.2%) 42 (41.8%)
Age in years	42.15 ± 5.84
Age at onset (years)	35.46 ± 3.96 (24–40)
Duration of disease (years) Family history of PD	6.68 ± 4.48 (1–19) 19.1%
Medications	
Levodopa	91%
Dopamine agonists Trihexyphenidyl COMT-I	95.5% 56.2% 1.1%
Daily levodopa dose in mg	405.80 ± 364.25
Hoehn and Yahr	2.63 ± 0.62
MDS – UPDRS	
Part I	10.08 ± 7.33
Part II	11.25 ± 6.29
Part III	36.00 ± 13.68
Part IV	
Motor fluctuation Dyskinesia	85.4% 30.3%

### 3.2 Non-motor symptoms

**Fig. 1:**
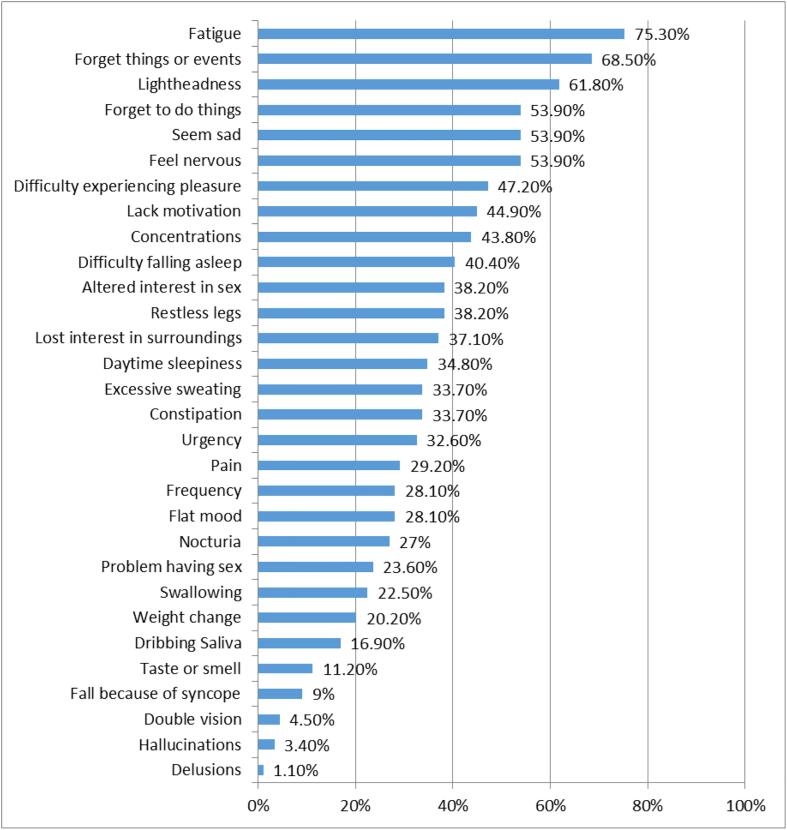
The frequency distributions of non-motor symptoms included in NMSS.

Study participants reported a mean ± SD of 10.17 ± 4.74 non-motor symptoms across 5.3 ± 1.7 domains of the NMSS. The median NMSS score was 37 (IQR 40). The prevalence and median (IQR) of each NMSS domain were: cardiovascular 62.9% and 1 (4); sleep/fatigue 89.9% and 8 (13); mood/cognition 83.1% and 6 (18); perceptual problems/hallucinations 6.7% and 0 (0); attention/memory 82% and 5 (8); gastrointestinal tract 49.4% and 0 (4); urinary 57.3% and 2 (7); sexual function 40.4% and 0 (4); miscellaneous 52 (58.4%) and 2 (8). The frequency distribution and severity of each non-motor symptom are shown in Fig. 1.

### 3.3 Quality of life in YOPD

The median (IQR) of PDQ-39SI score was 32.89 (16.8). The median (IQR) value of each dimension was: mobility 37.33 (21.96), ADL 42.93 (25.33), emotional well-being 39.77 (25.47), stigma 38.19 (28.44), social support 19.03 (22.89), cognition 29.59 (20.63), communication 26.96 (23.57), bodily discomfort 29.96 (23.19).

### 3.4 Correlations other factors and health related quality of life in YOPD

Pearson correlation coefficients showed statistically significant positive correlations between PDQ-39SI scores and disease duration r(df) = 0.216 (87), p = 0.045; UPDRS score part I r(df) = 0.532 (87), p < 0.001; part II, r(df) = 0.519 (87), p < 0.001; part III r(df) = 0.382 (87), p < 0.001; part IV r(df) = 0.254 (87), p = 0.016. Details of the univariate analysis were shown in Table 2. On the other hand, the Spearman rank correlation coefficients showed positive correlations of PDQ-39SI and scores of each NMSS domain including cardiovascular r(df) = 0.325 (87), p = 0.002, sleep/fatigue r(df) = 0.477 (87), p < 0.001, mood/cognition r(df) = 0.484 (87), p < 0.001, perceptual problems/hallucinations r(df) = 0.210 (87), p = 0.048, attention/memory r(df) = 0.325 (87), p = 0.002, urinary r(df) = 0.254(87), p = 0.016 and miscellaneous r(df) = 0.303(87), p = 0.004. The correlations of each NMSS domain and each PDQ-39 dimension are listed in Table 3.

**Table 2:** Correlations of HRQoL (PDQ-39) dimensions with demographic infomation, disease-related factors and motor manifestions (UPDRS).

		Mobility	ADL	Emotional well-being	Stigma	Social support	Cognition	Communi-cation	Bodily discomfort	PDQ39 - SI
Age of onset	r	0.01	0.192	−0.021	−0.084	−0.086	0.068	−0.83	0.008	−0.008
	*p*	0.991	0.072	0.845	0.434	0.421	0.528	0.439	0.942	0.941

Sex	r	0.18	−0.73	0.182	−0.033	0.025	−0.127	−0.048	−0.081	−0.017
	*p*	0.87	0.494	0.088	0.757	0.813	0.236	0.658	0.448	0.876

Hoehn & Yahr	r	**0.244**	**0.238**	0.098	0.043	0.119	0.012	**0.222**	0.134	0.201
	*p*	0.021	0.025	0.363	0.692	0.266	0.91	0.036	0.21	0.059

Disease duration	r	**0.314**	**0.289**	0.025	0.142	0.71	−0.028	**0.398**	−0.22	**0.216**
	*p*	0.003	0.006	0.817	0.186	0.509	0.796	0.000	0.836	0.042

Daily L-dopa dose	r	0.175	0.130	−1.26	0.031	−0.089	0.072	0.107	−0.057	0.042
	*p*	0.1	0.226	0.239	0.771	0.407	0.502	0.319	0.595	0.695

MDS-UPDRS part I	r	**0.4**	**0.406**	**0.375**	0.166	**0.368**	**0.442**	**0.331**	**0.564**	**0.532**
	*p*	0.0001	0.000	0.000	0.12	0.000	0.000	0.002	0.000	0.000

MDS-UPDRS part II	r	**0.554**	**0.539**	**0.295**	0.187	**0.342**	**0.28**	**0.401**	**0.372**	**0.519**
	*p*	0.0001	0.000	0.005	0.079	0.001	0.008	0.000	0.000	0.000

MDS-UPDRS part III	r	**0.235**	**0.399**	**0.328**	**0.251**	**0.259**	0.142	**0.269**	**0.279**	**0.382**
	*p*	0.027	0.000	0.002	0.018	0.014	0.183	0.011	0.008	0.000

MDS-UPDRS part IV	r	**0.302**	**0.282**	0.2	0.093	**0.211**	−0.17	**0.315**	0.013	**0.254**
	*p*	0.004	0.007	0.06	0.389	0.047	0.875	0.003	0.901	0.016

Total UPDRS	r	**0.498**	**0.596**	**0.455**	**0.284**	**0.426**	**0.310**	**0.459**	**0.467**	**0.615**
	*p*	0.0001	0.0001	0.0001	0.007	0.000	0.003	0.000	0.000	0.000

**Table 3:** Correlations of HRQoL (PDQ-39) dimensions with non-motor manifestations (NMSS domains).

		Mobility	ADL	Emotional well-being	Stigma	Social support	Cognition	Communi-cation	Bodily discomfort	PDQ39 - SI
Cardiovascular	**r** **p**	**0.285** 0.007	**0.260** 0.014	0.159 0.136	**0.236** 0.026	0.131 0.220	0.523 0.603	**2.206** 0.03	**0.258** 0.015	**0.325** 0.002
Sleep/Fatigue	**r** **p**	**0.399** <0.001	**0.34** 0.001	**0.357** 0.001	0.208 0.051	**0.291** 0.006	0.196 0.065	**0.300** 0.004	**0.474** <0.001	**0.477** <0.001
Mood/Cognition	**r** **p**	**0.291** 0.006	**0.324** 0.002	**0.563** <0.001	**0.286** 0.007	**0.406** <0.001	**0.418** <0.001	**0.313** 0.003	**0.356** 0.001	**0.484** <0.001
Perceptual problems/hallucinations	**r** **p**	0.086 0.421	0.096 0.372	0.73 0.496	0.140 0.191	**0.233** 0.028	**0.365** <0.001	**0.235** 0.027	0.197 0.064	**0.210** 0.048
Attention/Memory	**r** **p**	0.096 0.371	0.111 0.301	**0.275** 0.009	0.183 0.087	**0.306** 0.004	0.013 0.907	**0.322** 0.002	0.184 0.084	**0.325** 0.002
Gastrointestinal tract	**r** **p**	0.192 0.072	0.166 0.119	−0.062 0.566	−0.007 0.948	−0.038 0.723	**0.437** <0.001	**0.259** 0.014	0.112 0.297	0.121 0.258
Urinary	**r** **p**	**0.280** 0.008	**0.385** <0.001	0.063v0.56	0.088 0.412	0.114 0.288	0.156 0.145	0.135 0.209	0.198 0.062	**0.254** 0.016
Sexual function	**r** **p**	0.1630.127	01310.219	−0.0440.680	0.0000.998	0.0560.599	0.1000.352	0.1930.069	0.1990.062	0.1090.307
Miscellaneous	**r** **p**	**0.269** 0.011	**0.246** 0.02	0.192 0.071	0.141 0.188	0.192 0.072	**0.256** 0.016	0.172 0.108	**0.338** 0.001	**0.303** 0.004
Total NMSS	**r**	**0.462**	**0.445**	**0.416**	**0.254**	**0.320**	**0.437**	**0.409**	**0.476**	**0.546**
	**p**	<0.001	<0.001	<0.001	0.016	0.002	<0.001	<0.001	<0.001	<0.001

The step-wise multiple linear regression with PDQ – 39SI as the dependent variable was calculated to evaluate the independent effects of the potential factors including sex, age of onset, disease duration UPDRS part III, UPDRS part IV and scores of eight NMSS domains. The regression model consisting of NMSS domain 2 - sleep/fatigue, NMSS domain 3 - mood/cognition, NMSS domain 5 - attention/memory and UPDRS part III explained 43.3% of the variance of PDQ-39SI, F (4,84) = 17.778, p < 0.0005. There was no collinearity in the regression model as suggested by the variance inflation factor analysis. The final model revealed that crucial independent predictors of worsening HRQoL as measured by PDQ-39SI score were NMSS domain 2 - sleep/fatigue F(4,84) = 2.112 , p = 0.038; NMSS domain 3 - mood/cognition F(4,84) = 2.802, p = 0.006, NMSS domain 5 - attention/memory F(4,84) = 2.558 , p = 0.012 and MDS-UPDRS part III (motor symptoms) F(4,84) = 3.258, p = 0.002. Details of the multivariate analysis are illustrated in Table 4.

**Table 4:** Results of the stepwise multiple linear regression with PDQ-39SI as the dependent variable.

Model (Adj. R^2^ = 0.433)	Unstandardized coefficients		Standardized coefficients	t	Sig.	R^2^ change	Collinearity statistics	
	B	Std. Error	Beta				Tolerance	VIF
(Constant)	8.492	4.182		2.031	0.045			
Mood/Cogition	0.252	0.120	0.226	2.112	0.038	0.303	0.563	1.776
MDS-UPDRS part III	0.336	0.103	0.273	3.258	0.002	0.059	0.917	1.091
Sleep/Fatigue	0.535	0.191	0.274	2.802	0.006	0.054	0.673	1.486
Attention/Memory	0.579	0.226	0.227	2.558	0.012	0.042	0.817	1.225

## 4 Discussion

As is now widely accepted, non-motor symptoms are key components of PD in the premotor as well as the motor stages [30]. Although non-motor manifestations substantially affect quality of life of symptoms, they may remain unrecognized and untreated [31]. Prior research has confirmed that the NMSS scale is a reliable and useful tool to assess a wide range of non-motor symptoms in individuals with PD [27]. In agreement with earlier findings [32], our results showed that the non-motor manifestations were common in patients with YOPD. “Fatigue” was the most prevalent symptom, reported in 75.3% participants, followed by “forget things or events” and “lightheadedness”. Similarly, a previous study showed that fatigue can be experienced in up to 81% of PD patients [33]. Across different NMSS domains, a recent largre cross-sectional study assessing non-motor symptoms in multi-national PD patients revealed that the highest domain score was observed for domain 2 (Sleep/Fatigue) [34]. Moreover, the highest prevalences of both fatigue symptom and sleep/fatigue among NMSS domains were reported in prior studies on PD patients [21], [35]. In our study sample, sleep/fatigue was not only the most prevalent NMSS domain, but it was also the most severely affected one as well. A cohort study in the southeast Asian population showed that fatigue was also common even in PD patients with clinically stable motor symptoms [36].

All enrolled patients reported relatively poor HRQoL as demonstrated by high PDQ-39 score. The participants reported impairement in different dimensions of HRQoL, among which the most severely affected ones were ADL, mobility and emotional well-being. The significant contribution of non-motor burden to diminished HRQoL has been consistently reported in various studies performed on patients with more typical, later-onset PD [16], [24]. Whether patients with YOPD have worse quality of life than that of patients with LOPD is still debatable [37], [38]. YOPD patients are usually the breadwinners in their households and therefore may suffer from greater socioeconomic burden thanpatients with LOPD [39].

In this study, we used the disease-specific validated instruments to explore the relations between non-motor burden and HRQoL in YOPD patients. Non-motor manifestations had a negative impact on different aspects of quality of life demonstrated by moderate positive correlations observed between total NMSS score and each PDQ-39 dimension. Interestingly, mood/cognition could be considered as the most troublesome non-motor manifestation, with significant correlations found across all PDQ-39 dimensions. On the other hand, HRQoL (PDQ-39SI) positively correlated with 7 out of 9 NMSS domains (excluding gastrointestinal tract and sexual function domain).

Within the NMSS scale, mood/cognition and sleep/fatigue showed the strongest correlations with PQQ-39SI, in accordance with previous findings [21], [35], [40]. Prior research confirmed that the presence of fatigue was associated with poor quality of life in typical PD [41] as well as in early, de novo PD [42]. Additionally, several earlier papers indicated that poor sleep quality was significantly associated with worsening quality of life in patients with PD [43], [44]. The work of Mahale et al. elucidated that sleep disorder had a stronger influence on quality of life in LOPD, compared to that in YOPD [45]. Consistent with previous findings in the literature, our study showed that sleep disturbances along with fatigue, as included in sleep/fatigue domain, was not only correlated with but also significantly predictive of decrements of HRQoL in individuals with YOPD. In his review, Kuhlman et al. highlighted that non-motor symptoms including anxiety, depression, excessive daytime sleepiness, apathy, and impairment in activities of daily living closely related to poor HRQoL in patients with PD [46]. Several prior studies underlined low quality of life in PD patients with depression or anxiety [18], [43]. Mood disorder was one of the most prevalent NMSS domains in our YOPD patients, similar to what has been reported in previous studies on typical PD [47]. In agreement with many other studies, beside sleep/fatigue, mood/cognition and attention/memory along with motor burden measured by MDS-UPDRS part III were proved to be determinats of HRQoL in patients with YOPD [26]. In stepwise multiple linear regression, R^2^ change of mood/cognition, MDS-UPDRS part III, sleep/fatigue and attention/memory were 0.303, 0.059, 0.054 and 0.042 respectively. According to our data, mood/cognition was the predictor with the greatest impact on HRQoL, even surpassing motor burden. In their work, Huijuan Li et al. [48] indicated that the ability of the NMSS to predict HRQoL appeared to be more robust than that of the MDS-UPDRS part III. Contrary to earier results reported in the literature indicating that motor symptoms had a tremendous influence on the quality of life [49], [50], we found that the impact of motor manifestations on the quality of life is not prominent in young-onset patients, possibly because it is already the primary focus for them to control roublesome motor function to be able to function at work.

This study has some limitations. The Vietnamese versions of the scales have not been validated. Furthermore, this is a single-center, non-controlled observational study with methodological problems such as selection bias, information bias and confounding. Hence, future studies with prospective designs and control groups can give better understanding on the NMSS progression and the long-term impact of the non-motor burden on HRQoL.

## 5 Conclusions

Our work demonstrated that non-motor symptoms pertaining to sleep/fatigue, mood/cognition and attention/memory domain significantly contributed and independently predicted worse HRQoL in individuals with YOPD. This finding highlights the need for better evaluation and management of non-motor features in YOPD and the consideration of those symptoms as important therapeutic targets in this patient population.

### CRediT authorship contribution statement

**Tai Ngoc Tran:** Conceptualization, Methodology, Resources, Data curation, Validation, Visualization, Supervision, Project administration, Writing – original draft. **Uyen Ngoc Le Ha:** Data curation, Formal analysis, Resources, Investigation, Software, Validation, Writing – original draft. **Tuan Manh Nguyen:** Conceptualization, Methodology, Investigation, Formal analysis, Software. **Thuan Duc Nguyen:** Conceptualization, Methodology, Funding acquisition. **Khang Ngoc Chung Vo:** Investigation, Resources. **Thuong Huyen Dang:** Investigation, Resources, Visualization. **Paula Mai Phuong Trinh:** Investigation. **Daniel Truong:** Writing – review & editing, Funding acquisition.

## Declaration of Competing Interest

The authors declare that they have no known competing financial interests or personal relationships that could have appeared to influence the work reported in this paper.
